# What is the consistency between the results of needle biopsy and prostatectomy specimen pathology results? A pilot study

**DOI:** 10.3906/sag-2009-73

**Published:** 2021-06-28

**Authors:** Ömer Ozan YILDIZLI, İbrahim ÜNTAN, Deniz DEMİRCİ

**Affiliations:** 1 Department of Urology, Melikgazi Hospital, Kayseri Turkey; 2 Department of Urology, Training and Research Hospital, Ahi Evran University, Kırşehir Turkey; 3 Department of Urology, Faculty of Medicine, Erciyes University, Kayseri Turkey

**Keywords:** Accuracy, biopsy pathology, correlation, prostate cancer, prostatectomy pathology

## Abstract

**Background/aim:**

The aim of this study was to establish the relationship between the needle biopsy and the pathology result after radical prostatectomy administrated for prostate cancer.

**Materials and methods:**

We retrospectively analyzed 67 patients who had undergone radical prostatectomy from 2016 to 2019. All surgeries and all biopsies were performed in the third author’s urology department. Samples were collected through 12-core biopsy under local anesthesia. All specimens were studied in the pathology department of the third author’s center. The results evaluated were needle biopsies’ Gleason scores and prostatectomy specimens’ Gleason scores.

**Results:**

Inclusion criteria were not having any neo-adjuvant treatment and being treated with surgery after needle biopsy. Gleason scores obtained from needle biopsies and prostatectomy specimens were evaluated. The comparison revealed that 39% of the tumors were undergraded, 7% were overgraded, and 54% had exact scoring in needle biopsies and prostatectomy specimens according to the detailed Gleason scoring as primary and secondary metrics. The patients were grouped into five categories according to the ISUP 2014 prostate cancer grading system. The relationship was strong with 64% of results staying in the same group after the operation; nevertheless, the correlation remained weak based on the kappa coefficient.

**Conclusion:**

The information obtained from the needle biopsy is not a strong herald of the pathological result. Urologists should have awareness of this restraint when utilizing the needle biopsy’s Gleason score in decision making and treatment planning.

## 1. Introduction

Clinical staging of the prostate cancer is essential in starting a therapy plan [1]. Transrectal ultrasound (TRUS)-guided prostate biopsy is a standard procedure for predicting postoperative pathological grade in many centers [2]. Clinical grades refer to prostate biopsy and are critical in patients who are candidates for radiotherapy or watchful waiting because these stages guide prognosis and treatment [3]. 

The accuracy of needle biopsy is inconclusive [4,5]. Magnetic resonance imaging (MRI)-guided prostate biopsy including MRI/ultrasound fusion biopsy is rapidly increasing with good accuracy [6,7]. On the other hand, repeat biopsy procedure and saturation biopsy methods can empower urologists to improve the accuracy [8,9]. Nevertheless, urologists still rely on 12-core needle biopsies because other methods are rare, require more personnel, and are more invasive. Therefore, we evaluated the correlation between the Gleason scores on biopsies and prostatectomy specimens in 67 patients who were diagnosed with prostate adenocarcinoma, had no neo-adjuvant treatment or radiotherapy, and had undergone radical prostatectomy (RP).

## 2. Materials and methods

The retrospective study was conducted in Erciyes University, Faculty of Medicine, Department of Urology. Sixty-seven patients who underwent RP for prostate adenocarcinoma in our center between 2016 and 2019 were enrolled. The patients’ files, charts, and computerized data were collected, and the pathology results of needle biopsies and prostatectomy specimens, ages, and PSA levels before biopsies were noted. Pathology results were summarized including Gleason score as the primary and secondary grading in needle biopsies and prostatectomy specimens, border extension, and seminal vesicle invasion in prostatectomy specimen results as well. Patients were subjected to clinical tumor node metastasis classification for staging (Table 1). T-staging is mainly based on digital rectal examination (DRE), but transrectal ultrasonography (TRUS) or multiparametric MRI is also considered if performed. N-staging was mostly done with computed tomography (CT) and MRI. M-staging was mostly done with bone scan. 

**Table 1:** Clinical tumor node metastasis classification of prostate cancer.

T- primary tumor		
TX	Primary tumor cannot be assessed	
T0	No evidence of primary tumor	
T1	Tumor that is palpable and confined within the prostate	
	T1a	Tumor incidental histological finding in 5% or less of tissue resected
	T1b	Tumor incidental histological finding in more than 5% of tissue resected
	T1c	Tumor identified by needle biopsy (e.g., because of elevated PSA)
T2	Tumor that is palpable and confined within the prostate	
	T2a	Tumor involves one half of one lobe or less
	T2b	Tumor involves more than half of one lobe, but not both lobes
	T2c	Tumor involves both lobes
T3	Tumor extends through the prostatic capsule	
	T3a	Extracapsular extension (unilateral or bilateral)
	T3b	Tumor invades seminal vesicle(s)
T4	Tumor is fixed or invades adjacent structures other than seminal vesicles: external sphincter, rectum, levator muscles, and/or pelvic wall	
N – regional (pelvic) lymph nodes		
NX	Regional lymph nodes cannot be assessed	
N1	No regional lymph node metastasis	
N2	Regional lymph node metastasis	
M - distant metastasis		
M0	No distant metastasis	
M1	Distant metastasis	
	M1a	Non-regional lymph node(s)
	M1b	Bone(s)
	M1c	Other sites(s)

Patients were defined as localized and locally advanced prostate cancer according to the PSA level, Gleason score, and clinical staging. For surgical treatment, a life expectancy of at least 10 years is required. Active surveillance, radiotherapy, and radical prostatectomy options are offered to the patients in the localized group. Radiotherapy and radical prostatectomy options are offered to patients in the locally advanced group (Table 2). The patients in our study are patients who proceeded according to this scheme and finally decided and applied radical prostatectomy. A senior surgeon managed all the RPs and another experienced surgeon helped the senior surgeon. 

**Table 2:** Risk groups for biochemical recurrence of localized and locally advanced prostate cancer.

Definition			
Low-risk	Intermediate-risk	High-risk	
PSA < 10 ng/mL	PSA 10 - 20 ng/mL	PSA > 20 g/mL	any PSA
and ISUP grade 1	or ISUP grade 2–3	or ISUP grade 4–5	any ISUP grade
and cT1 - T2a	or cT2b	or cT2c	cT3–4 or cN1
Localized			Locally advanced

All specimens were studied in our hospital’s own pathology department by randomly assigned pathologists. Clinical information (e.g., age, DRE, PSA) was also included. A positive surgical margin was defined as the presence of cancerous tissue in contact with the inked surface of the prostatectomy specimen. Healthy tissue margins were considered negative margins. Some of the biopsy reports where the tumor was graded as good, moderate, or poor differentiation did not contain Gleason scores; thus, these documents were excluded from the study. No patient received radiotherapy or hormone therapy before RP. The Gleason grades were compared separately in biopsy and pathology groups as primary and secondary. The sums of Gleason scores were compared in biopsy and pathology groups. Grade groups in which the score totals correspond to International Society of Urological Pathology (ISUP) were also compared before and after RP (Table 3) [10].

**Table 3:** ISUP 2014 grades.

Gleason score	ISUP grade
2–6	1
7 (3 + 4)	2
7 (4 + 3)	3
8 (4 + 4 or 3 + 5 or 5 + 3)	4
9–10	5

IBM SPSS version 22.0 (IBM Corp. Armonk, NY, USA) was used for statistical analysis of the datasets. Cohen’s kappa (κ) analysis was used to determine the agreement between biopsy and pathology results. The strength of the agreement was evaluated according to κ coefficient (Table 4). In all tests, the statistical significance level was set at 0.05.

**Table 4:** Kappa coefficient interpretation.

Value of κ	Strength of agreement
0	Chance agreement
<0.4	Poor agreement
0.4–0.75	Good agreement
<0.75	Excellent agreement

## 3. Results

The mean age of the patients was 60.4 ± 5.7 years (range: 46–78). The mean of the PSA levels was 11.5 ± 7.8 ng/mL (range: 4–54). The mean Gleason score was 6.25 ± 0.97 (range: 4–9) on needle biopsies and 6.69 ± 1.17 (range: 4–10) on pathology specimens (Table 5). 

**Table 5:** Patients’ descriptives.

n = 67			Gleason score on	
	Age	PSA level	Biopsy	Specimen
mean	60.4	11.5	6.25	6.69
sd ±	5.7	7.8	0.97	1.17
range	46–78	4–54	4–9	4–10

The biopsy results were described as primary and secondary Gleason grades and were compared to the results of the prostatectomy specimens; Gleason grades on biopsies remained identical in 54%, were undergraded in 7%, and overgraded in 39% on prostatectomy specimens. The κ coefficient was calculated as 0.37 in statistical correlation tests (p < 0.01). The primary and secondary Gleason grades of the biopsy revealed a poor prediction of prostatectomy specimens’ primary and secondary Gleason grades.

The biopsy results were defined as the individual Gleason scores and confirmed with prostatectomy specimens’ results. The total Gleason scores of biopsies remained the same in 55%, were undergraded in 9%, and overgraded in 36% on prostatectomy specimens. The κ coefficient was 0.34 in statistical correlation tests (p < 0.01). The individual Gleason score of the biopsy showed a poor prediction of prostatectomy specimens’ individual Gleason scores.

The results were divided into groups according to ISUP 2014 prostate cancer grading system. The biopsies were compared with the groups of prostatectomy specimens, and the groups of biopsies stayed the same in 64%, undergraded in 8%, and overgraded in 28% on groups of prostatectomy specimens. Although the number that remained identical increased, this was not reflected in the accuracy test. The κ coefficient was computed as 0.39 in statistical correlation testing (p < 0.01). The biopsy groups in binary groups had a poor correlation with prostatectomy specimens (Table 6).

**Table 6:** Overall correlation for ISUP grades in the biopsy and prostatectomy specimens.

(Total = 67)	ISUP grade on prostatectomy specimen				
ISUP grade on biopsy	Grade 1(n = 34)	Grade 2(n = 15)	Grade 3(n = 8)	Grade 4(n = 3)	Grade 5(n = 7)
Grade 1 (n = 44)	30	8	3	2	1
Grade 2 (n = 13)	3	7	1	0	2
Grade 3 (n = 5)	1	0	3	0	1
Grade 4 (n = 3)	0	0	1	1	1
Grade 2 (n = 2)	0	0	0	0	2

## 4. Discussion

Prostate cancer ranks fourth among cancers worldwide [11]. DRE is a simple and established tool to diagnose prostate cancer [12]. Current methods can provide a cure to suitable patients via radical prostatectomy [13]. Patients with localized prostate cancer who do not have prostatic capsule involvement and have no evidence of metastasis are suitable for radical prostatectomy [14]. However, radical prostatectomy is not performed in every patient with localized prostate cancer [15]. Clinical staging is decisive when planning the treatment of patients with prostate cancer [16]. Depending on the clinical stage, watchful waiting or radiation therapy may also be an alternative [17]. Thus, special efforts have been made for accurate clinical staging. The most important condition of correct clinical staging is that the biopsy results show the pathology results with high accuracy. 

The most common mismatch between biopsy and pathology in the literature is the undergrading of biopsy [18]. We found that biopsy samples showed undergrading versus pathology in each evaluation group. In addition, the prostate biopsy does not have sufficient accuracy, which is a common issue in the literature [19]. In the literature, multiparametric prostate MRI and MRI fusion biopsy have higher kappas than conventional prostate biopsy [20,21] 

In the literature, studies reporting a weak correlation between biopsy and prostatectomy total Gleason scores are common [22]. We found that the compliance was 55% with weak correlation when the total Gleason score was considered. Similarly, the literature shows discrepancy when the Gleason score was defined as the primary and secondary points [23]. There was 54% agreement and weak correlation consistent with the literature. In our study, the total Gleason scores were classified according to ISUP 2014 prostate cancer grading system, and the agreement was 64% with correlation statistics that showed poor agreement. This result was compatible with the literature [24]. In detail, in the grade 2 group, 54% remained identical, 23% were undergraded, and 23% were overgraded. In grade 3, 60% remained identical, 20% were undergraded, and 20% were overgraded. With a more accurate method, about a quarter of these patients could have been kept under active surveillance and thus could have been protected from the side effects of the treatment if only temporarily. 

Accuracy and correlation are important in common malignant disease and staging is important. Our data and the literature suggest that only conventional biopsy is insufficient to predict pathology results. In light of this information, prostate biopsy should not be trusted alone. Considering that there are as many undergraded results as accurate results, treatment planning based on these results alone may not be accurate. We found that prostate biopsy is not so reliable as to ignore alternative methods. Urologists should try to compensate for this limitation by using repeat biopsy, saturation biopsy, and/or MRI-supported biopsy according to the case history and details.

## Ethics approval and consent to participate

All procedures performed in human participants were in accordance with the ethical standards of the institutional and/or national research committee and with the 1964 Helsinki Declaration and its later amendments or comparable ethical standards. This study was conducted under the approval of the Ethical Council of Erciyes University, Faculty of Medicine (13.12.2013 / 98681246-340). Informed consent was obtained from all individual participants included in the study.

## Patient consent for publication

All patients gave informed written consent about being a part of this clinical research and publication.

## Authors’ contributions

İÜ and ÖOY conceived and designed the study. ÖOY collected the data and İÜ set up the study, analyzed the data and wrote the manuscript. DD provided supervision.
